# Relationship between clinical presenting patterns of acute myocarditis and oedema and late enhancement extension

**DOI:** 10.1186/1532-429X-14-S1-P185

**Published:** 2012-02-01

**Authors:** Alberto Roghi, Daniela Lanza, Patrizia Pedrotti, Angela Milazzo, Ornella Rimoldi, Stefano Pedretti

**Affiliations:** 1 IBFM Centro PET Settore C, CNR, Milano, Italy; 2 CMR Unit Dept of Cardiology, Niguarda Ca’Granda Hospital, Milan, Italy; 3 Cardiology, University of Verona, Verona, Italy

## Summary

Acute myocarditis clinical onset can span from subclinical disease to acute heart failure, fatal arrhythmias or sudden cardiac death. Patients with more severe clinical onset have larger areas of inflammation and contrast enhancement which are directly correlated with left ventricular systolic function.

## Background

acute myocarditis (AM) clinical onset can span from subclinical disease to acute heart failure (AHF) ventricular fibrillation (VF) or sudden cardiac death in young adults. Myocarditis can underly the aetiology of other cardiomyopathies (CM) such as dilated CM arrhythmogenic left or right ventricle CM. Aim of the study was to evaluate the relationship between myocardial oedema and late enhancement (LGE) extension and clinical presenting patterns of acute myocarditis by means of cardiac magnetic resonance imaging (CMR).

## Methods

Eighty-two consecutive patients (pts) referred for suspected myocarditis from 2007 to 2010 were retrospectively analyzed. Symptoms, ECG changes, reduced myocardial function, elevated creatine kinase, positive troponin T, suggested AM. Coronary artery disease was excluded at angiography. Patients were studied on days x±y after the onset of symptoms The diagnosis was confirmed by CMR (Siemens Avanto 1.5 Tesla) according to the presence of typical signal hyperintensity at Short Tau Inversion Recovery (STIR) images, associated with concordant LGE (0.1mmol/Kg gadobutrol) distribution . The area of enhancement on STIR and CE-IR images were measured by commercial software and expressed as percentage of the LV. Data are x±SD, significant difference p<0.05.

## Results

According to the initial clinical picture pts were divided into two groups: group 1 (G1;n= 68), presenting with chest pain; group 2 (G2; 14 pts), presenting with AHF or VF. Haemodynamic and functional parameters were similar in the 2 groups (Table), in G2 EF was slightly lower. G2 showed a larger area of oedema on STIR images and LGE distribution. LVEF was significantly correlated both to STIR (R= 0.49 p<0.0001) and LGE (R= 0.4 p<0.0006) percentages.

**Table 1:** 

	Group 1	Group 2	p
Age	31 ± 11	38 ± 11	0.046
Female	13%	29%	0.15
LV EDV (ml/m2)	76 ± 15	71 ± 17	0.20
LVESV(ml/m2)	28 ± 10	30 ± 13	0.5
LV EF(%)	64 ± 8	58 ± 14	0.13
LV mass (g)	148 ± 36	164 ± 44	0.155
RV EF(%)	62 ± 6	61 ± 11	0.662
STIR+ percentage (%)	9 ± 7	33 ± 23	<0.0001
LGE percentage (%)	8 ± 6	19 ± 20	0.047
Pericardial effusion	29%	50%	0.136

## Conclusions

Pts with AM presenting with AHF or VF at admission showed significantly larger percentage of oedema and of LGE. Our data suggest a direct relationship between the severity of clinical presentation and the extension of myocardial damage.

## Funding

Department of Cardiology, Niguarda Ca’Granda Hospital, Milan, Italy.

